# Health and Wellbeing Benefits from Nature Experiences in Tropical Settings Depend on Strength of Connection to Nature

**DOI:** 10.3390/ijerph181910149

**Published:** 2021-09-27

**Authors:** Rachel R. Y. Oh, Kelly S. Fielding, Chia-Chen Chang, Le T. P. Nghiem, Claudia L. Y. Tan, Shimona A. Quazi, Danielle F. Shanahan, Kevin J. Gaston, Roman L. Carrasco, Richard A. Fuller

**Affiliations:** 1 Centre for Biodiversity and Conservation Sciences, School of Biological Sciences, University of Queensland, Brisbane 4072, Australia; r.fuller@uq.edu.au; 2 Department of Ecosystem Services, Helmholtz Centre for Environmental Research (UFZ), 04318 Leipzig, Germany; 3 German Centre for Integrative Biodiversity Research (iDiv) Halle-Jena-Leipzig, 04103 Leipzig, Germany; 4 School of Communication and Arts, University of Queensland, Brisbane 4072, Australia; k.fielding@uq.edu.au; 5 Department of Biological Sciences, National University of Singapore, Singapore 117558, Singapore; chiajen.chang@gmail.com (C.-C.C.); nghphuongle@nus.edu.sg (L.T.P.N.); claudiatan_ly@u.nus.edu (C.L.Y.T.); dbsctlr@nus.edu.sg (R.L.C.); 6 National Parks Board, Singapore 259569, Singapore; Shimona_QUAZI@nparks.gov.sg; 7 Zealandia Centre for People and Nature, Wellington 6012, New Zealand; danielle.shanahan@visitzealandia.com; 8 Environment & Sustainability Institute, University of Exeter, Cornwall TR10 9FE, UK; K.J.Gaston@exeter.ac.uk

**Keywords:** urbanisation, public health, health and wellbeing, nature exposure, nature experiences, nature dose

## Abstract

A growing number of policies and programmes in cities aim to increase the time people spend in nature for the health and wellbeing benefits delivered by such interactions. Yet, there is little research investigating the extent to which, and for whom, nature experiences deliver such benefits outside Europe, North America, and Australia. Here, we assessed the relationships between nature dose (frequency, duration, and intensity) and three mental wellbeing (depression, stress, and anxiety) and two physical health (high blood pressure, diabetes) outcomes in Singapore, an intensely urbanised tropical city. Our analyses accounted for individual factors, including socio-economic status, nature connection (nature relatedness), and whether people with poor health are prevented by their condition from visiting green spaces. Our results show that the association between nature dose (specifically duration) and mental wellbeing is moderated by a nature connection. Specifically, people with a stronger nature connection were less likely to be depressed, stressed, and anxious, regardless of the duration of their nature dose. For those with a weaker connection to nature, spending longer in nature was associated with being more depressed, stressed, and anxious. We did not find a relationship between nature dose and high blood pressure or diabetes. Our results highlight that the relationship between nature dose and wellbeing might vary substantially among cities.

## 1. Introduction

Urbanisation has emerged as one of the most important global human health challenges of the 21st century [1,2]. While urban residents are on average wealthier and receive better nutrition and healthcare than rural dwellers [3], urban living has also been associated with increased risk of chronic disorders, a more demanding and stressful social environment, and greater social disparity [4,5]. Cities are becoming epicentres for chronic, non-communicable physical and mental health conditions [6,7,8], and meta-analyses have shown that people in urban settings have a substantially increased risk for anxiety, mood, and brain disorders [7,8,9]. Other urban health risks include pandemic outbreaks (e.g., SARS-CoV-2 virus [10]), exposure to noise, water and air pollution, contagious diseases related to higher population density (e.g., tuberculosis), and risks associated with homelessness, violence, and inequality [11].

Exposure to nature has been proposed as a tool for providing health and wellbeing benefits in urban environments [12]. For example, health practitioners are increasingly prescribing nature-based experiences for patients living with specific health conditions [13,14], while non-government organisations (NGOs) have lobbied for 1% of all health expenditure to be invested in nature-based solutions [15]. Indeed, there is a growing body of epidemiological evidence indicating that urban green spaces play a crucial role in addressing public health challenges [16,17,18]. While the quantity and quality of evidence varies across health outcomes, greater exposure to, or contact with, natural environments such as parks and forests has generally been associated with improved physical health (e.g., reduced blood pressure [19,20]; fewer allergies [21]), better self-reported general health [22,23], higher subjective wellbeing (e.g., reduced stress [24]; improved self-esteem and mood [25]; improved restoration [26,27]) and lower probability of mortality among adults [22,28]. In fact, the “dose” of nature exposure (e.g., frequency and duration of green space visits) is positively correlated with some of the benefits gained [29], and it has been estimated that up to 27% of depression cases could be prevented by spending five or more hours per week in a garden [30]. These findings highlight the possibility that exposure to nature makes a measurable contribution to health, and that urban green spaces may constitute a powerful public health intervention. 

However, an urban environment comprises not only natural green spaces but also built (e.g., pedestrian sidewalks, buildings) and social (e.g., interactions between urban residents) components [31] that may have complex impacts on health and wellbeing outcomes. For example, some studies have reported that greater population densities are associated with better health and wellbeing outcomes, as densely populated urban neighbourhoods have infrastructure that encourages active modes of transport, such as walking [32], which are protective against cardiometabolic disease risk [33,34,35]. Cross-sectional studies from North America have shown that densely populated areas are associated with lower prevalence of obesity and type-2 diabetes [36,37]. Yet, urban living also brings its own risk factors for mental health. People living in urban areas have higher rates of psychiatric morbidity [38] and are at substantially increased risk of anxiety and mood disorders [8]. The incidence of schizophrenia, a major brain disorder, doubles in individuals born and raised in cities, with evidence of a dose–response relationship that probably reflects causation [6,7]. Yet, current population-level knowledge on relationships between every day nature exposure and health outcomes is predominantly shaped by research on and from cities in North America, Europe, and Australia. This is despite the fact that cities vary globally in many ways such as the amount and spatial configuration of urban green spaces [39], governmental policies and planning [40], local and regional climates within which cities are situated [41], socio-economic conditions and demography [42], and human behaviour, perceptions, and values [43,44]. For example, there is considerable variability in peoples’ connection to nature, i.e., the extent to which people are attracted to nature. This human–nature relationship is multidimensional, consisting of affect (feelings towards nature), cognition (knowledge and beliefs about nature), and behaviour (actions and experiences in nature) [45]. These differences therefore raise the question of whether the health benefits from exposure to nature might vary, and to what extent, for cities beyond North America, Europe, and Australia.

To address this question, we conducted a cross-sectional study of urban dwellers in Singapore, a densely occupied tropical city. We measured three aspects of nature dose (i.e., direct exposure to nature [46])—the *frequency* and *duration* of a person’s everyday nature exposure, and the *intensity* (i.e., the quality and quantity) of that exposure. We focused on three mental wellbeing (depression, anxiety, and stress) and two physical health (high blood pressure and diabetes) outcomes, as these are pertinent to living in cities and there is evidence that nature exposure confers some protection against poor outcomes [47]. Most studies have assumed that nature exposure results in positive health outcomes, but have been unable to exclude potential reverse causality in which people with poor health may be less likely to spend time in nature because of their health condition. Our study attempts to account, in part, for this possibility by asking people whether poor health prevents them from visiting urban green spaces; we then include this as a variable in our statistical analyses.

## 2. Materials and Methods

Study Site—We chose Singapore as a study site as it is a highly urbanised and densely populated tropical city. The city-state has a total land area of 724 km^2^ [48], with a population of 5.7 million residents [49]. While the city-state has an average population density of 7804 residents per square kilometre, the urban population density (i.e., ratio of the total urban population of the city and its urban area) can reach 18,600 residents per square kilometre [50]. This is much higher than average urban population densities in European (e.g., London: 6300 residents/km^2^, Berlin: 5600 residents/km^2^), American (e.g., New York: 3300 residents/km^2^, Los Angeles: 3300 residents/km^2^), and Chinese (e.g., Beijing: 7800 residents/km^2^, Shanghai: 7600 residents/km^2^) cities [50].

*Survey*—We delivered an online survey to Singapore residents within a one-month window in January 2019 through a market research company to a stratified subset of adults (18 years and above) voluntarily enrolled in their survey database. This survey was conducted in accordance with the University of Queensland Institutional Human Research Ethics Approval (project number 2018001775) and the Institutional Review Board at the National University of Singapore (project reference S-18-344). Informed consent was obtained from all respondents through a tick-box. Respondents were stratified by several nested criteria to ensure that the final sample was representative of the Singapore population in terms of standard demographic characteristics, with an even spatial distribution across the city. The stratification criteria were—(i) gender (50% males, 50% females); (ii) age (50% aged 18 to less than 45 years old, 50% aged 45 to 75 years old); (iii) personal income (reflecting the four quartiles from the national census); and (iv) greenery surrounding current residence (reflecting the four quartiles of tree cover across the city).

Exposure to nature—We measured three aspects of exposure to nature, namely the duration and frequency of nature experiences, and nature intensity through a mixture of self-reported and remote sensing analysis.

Respondents first reported on the duration of “outdoor green space” visit(s). Green space was introduced as: “For example, this includes beaches, parks and nature reserves, rooftop gardens, golf courses and gardens.” The estimated average duration of one green space visit was based on the self-reported number of hours spent during each visit in the previous week. We chose this timeframe as it provided a short and recent reference period to improve recall accuracy [51]. We summed the total number of hours spent across all visits in the previous week and capped it at 34 h, as 98% of the respondents reported spending a total of ≤34 h on green space visits. We then averaged the total duration by the number of green space visits in the previous week.

Respondents also reported the estimated frequency of regular green space visits in the past year. This was capped at 300 visits to avoid exaggerated positive skews in the frequency estimates, as 98% of respondents reported ≤300 visits in the past year. Compared to the duration measurement, we chose a different timeframe to allow us to account for people who visited green spaces infrequently, such as those who visit green spaces less than once a week and would not have been captured by the above duration measure, as well as to minimise correlation with the duration measure.

We also generated two measures of nature intensity, and this was made possible as respondents had provided the name of green spaces visited in the previous week. Nature intensity refers to the vegetation complexity within visited green spaces. It was chosen as it is an indicator of ecological complexity, and higher ecological complexity has been correlated with higher levels of biodiversity [52,53,54] and could lead to improved health outcomes [55]. We first geolocated the green spaces by aligning the provided names with the established spatial boundaries as per the Singapore Masterplan 2019 [56] in ArcGIS. We then overlaid a classified map of terrestrial ecosystems in Singapore, comprising four landcover categories (i.e., vegetation with limited human management [with Tree Canopy]; vegetation with structure dominated by human management [with Tree Canopy]; freshwater swamp forest; mangrove forest) with a 5 × 5 m resolution [57]. Following this, we generated two measures of nature intensity to represent the possible multiple pathways linking nature and health outcomes [29]—(i) the average area of tree cover within the largest vegetated green space visited by each respondent (quantity); and (ii) the proportion of the average area of tree cover that is managed by humans (quality). The quantity was derived using the spatial boundaries delineating each park, while the quality of green space was calculated by dividing the area of “vegetation with structure dominated by human management (with tree canopy)” by the quantity of green space as computed above. We included the second variable to differentiate between tree cover that is natural and unmanaged from tree cover that is managed, as areas in Singapore with high natural greenery are likely to be more biodiverse than those that are highly managed [58]. Smaller values of quality of green spaces indicated less complex vegetation. Measures involving nature intensity were limited to larger urban green spaces such as parks and nature reserves (rather than community and rooftop gardens) as these could be geo-located through the provided names and had established spatial boundaries as per the Singapore Masterplan 2019 [56].

Heath outcomes—Respondents provided information on three dimensions of mental wellbeing (i.e., depression, anxiety, and stress) and two physical health (high blood pressure and diabetes) outcomes. For mental wellbeing, we generated measures of depression, anxiety, and stress (experienced in the past week) using the Depression, Anxiety and Stress Scale (DASS21 [59]). Respondents rated a set of 21 statements with four responses ranging from “0 = Not applicable to me”, “1 = Applicable some of the time”, “2 = Applicable for a good part of time”, and “3 = Applicable for most of the time”. Within the set of 21 statements, 3 sets of 7 statements measured depression, anxiety, and stress, respectively. With regard to physical health, respondents reported whether they were currently receiving treatment for high blood pressure or diabetes. This was coded as a binary variable (0 = not receiving medical treatment; 1 = receiving medical treatment).

Covariates—We collected socio-demographic covariates including age, personal income, gender, ethnicity, highest formal education, and number of workdays per week, as these have been tied to health outcomes and experiences of nature in previous studies [28,29,60,61,62,63]. We also measured an individual’s connection to nature using the nature relatedness scale [64], where respondents were invited to rate a set of 21 statements using a five-point Likert scale ranging from 1 (disagree strongly) to 5 (agree strongly). An aggregation of the responses (as per [64]) provided a measure of an individual’s relationship with nature, with a higher score indicating a stronger connection with nature. Although the three sub-scales can be combined to form a unidimensional scale, we retained the three separate established sub-scales, and used two in our study—(i) *NR-Self (affective)*, which assesses how strongly one identifies with nature (e.g., my relationship to nature is an important part of who I am); and (ii) *NR-Experience (experiential)*, which indicates one’s physical familiarity, and attraction, with nature (e.g., I enjoy being outdoors, even in unpleasant weather [64]), as these sub-scales are likely to differentiate between groups of individuals who engage with nature to a greater or lesser extent. We additionally controlled for whether poor health prevented respondents from spending time in outdoor green spaces. This binary covariate comprised two groups of respondents—those who had indicated (i) “often” and “most of the time”; or (ii) “never” and “sometimes”, when asked about the extent to which “poor health” prevents them from spending time in an outdoor green space.

*Statistical Analyses*—We conducted all analyses in R version 3.6.2 [65]. We constructed a total of five global models to examine the correlation between each health response and potential predictors. We used cumulative link mixed models (CLMM) for depression, anxiety, and stress (*ordinal* package [66]) because the response variable (i.e., rating of each statement) is an ordered factor variable, and generalised linear models (binomial) for both high blood pressure and diabetes, because the response variable is a binary variable.

The predictor variables specified for the models are summarised in Table 1. All continuous predictors were standardised. We also included four interaction terms (duration and frequency of green space visit with each nature relatedness subscale), as the association between nature dose and health may be dependent on one’s connection to nature (nature relatedness). A Hessian condition number was used to assess goodness-of-fit for CLMMs [67], while residuals of fitted models were assessed for generalised linear models. Prior to all analyses, we assessed multicollinearity in each global model using the *vif* function from the *car* package [68], and found no such issues (VIF < 3).

We further conducted two types of sensitivity analyses. First, we analysed the depression, anxiety, and stress data using generalised negative binomial linear models (*MASS* package [69]) instead of CLMMs, as it was possible that a different treatment of the response variable (from ordinal to continuous) might result in a different set of significant predictors between model types. For this sensitivity analysis, we therefore aggregated the responses to form a continuous score that provided a measure of an individual’s extent of depression, anxiety, and stress, respectively, with a higher score indicating more severe depression, anxiety, and stress. The significant parameters were very similar to the CLMM analyses and are reported in the Supplementary Materials S1 (Tables S1–S4). For the second sensitivity analysis, we analysed only the depression and high blood pressure data by emulating as closely as possible the statistical methods used by Shanahan et al. [29], as they measured these health outcomes and predictor variables using very similar questions. Specifically, we converted the aggregated depression score into a binary measure where 0 indicated no depression (i.e., aggregated scores of 0–4; as per Shanahan et al. [29]) and 1 indicated mild or worse depression (i.e., aggregated scores of ≥5). We did so for two reasons: it was possible that (i) a different treatment of the depression response (from ordinal to binary); and (ii) the specification of four additional interaction terms between nature-relatedness and duration and frequency of green space used could result in a different set of significant predictors. We report these results in the Supplementary Materials S2 (Table S5) given that the significant predictors and direction of relationships between significant predictors were generally consistent with the CLMM analyses.

Lastly, we analysed whether individuals whose poor health prevented them from accessing outdoor green spaces were associated with any specific socio-economic factors by constructing a generalised linear (binomial) model. The binary response variable was whether the respondent indicated that poor health prevented them from spending time in outdoor green spaces, and the predictor variables were age, income, gender, education, and ethnicity.

## 3. Results

We received a total of 1499 responses to the urban lifestyle survey in Singapore. The respondent pool closely reflected the intended stratification criteria. The ethnic composition of the respondents approximated that of the national population (i.e., 70% Chinese, 15% Malay, 7% Indian, and 8% Others [70]).

### 3.1. Mental Wellbeing Outcomes

**Figure 1:**
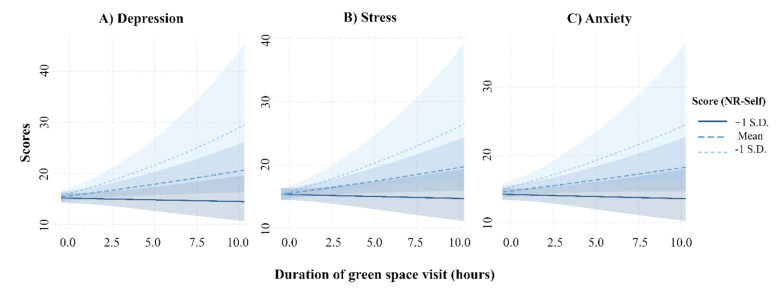
Interaction plots from the mixed-effects model showing the relationship between duration of nature interaction with each mental wellbeing score—(**A**) depression; (**B**) stress; and (**C**) anxiety. Fitted lines represent the mean, +1 standard deviation (S.D.), and −1 standard deviation scores for ecological identity (i.e., nature relatedness—self). Shaded area represents confidence intervals.

Overall, we found that nature dose (i.e., frequency, duration, and intensity) was not categorically associated with positive health and wellbeing outcomes. Instead, the strength of nature connection, in particular NR-Self, moderated the relationship between the duration of green space visits and all three mental wellbeing outcomes (Table 2). People with higher NR-Self were less likely to show symptoms of depression, stress, and anxiety, regardless of the duration of their exposure to nature. Conversely, for those with lower NR-Self, longer durations of nature exposure were correlated with higher levels of depression, stress, and anxiety (Figure 1). We also found that people whose health did not impede their use of green spaces were less likely to show symptoms of depression, stress, and anxiety (Table 2). Being older and non-Chinese was significantly associated with lower levels of depression, stress, and anxiety (Table 2). These results were comparable with the results from the two sensitivity analyses (reported in the Supplementary Materials).

There were also slight variations in the factors that were significantly associated with each mental wellbeing outcome. Lower levels of depression were significantly associated with stronger feelings of social cohesion (Table 2; Supplementary Materials S1. Lower levels of stress were significantly associated with higher personal income and less exercise (Table 2), while lower levels of anxiety were significantly associated with more frequent green space visits and less exercise (Table 2).

### 3.2. Physical Health Outcomes

With regard to physical health outcomes, 10.7% (n = 160) and 4.3% (n = 64) of all respondents (n = 1499) were receiving medical treatment for high blood pressure and diabetes, respectively. Both age and BMI were significantly and positively associated with the likelihood of receiving medical treatment for high blood pressure and diabetes (Table 3). There was no significant association between any measures of exposure to nature (and the specified interactions with connection to nature) and the likelihood of receiving treatment for high blood pressure or diabetes (Table 3). There were, however, slight variations in the factors that were significantly associated with each physical wellbeing outcome. Individuals whose health did not impede their use of green spaces were less likely to be receiving medical treatment for high blood pressure. Less exercise but stronger feelings of social cohesion was associated with a higher likelihood of receiving treatment for diabetes (Table 3).

Overall, 14% of respondents (n = 212) were unable to access outdoor green spaces because of poor health; they were generally older people (Table 4).

## 4. Discussion

In this context of a densely populated, highly urbanised, multicultural, and tropical city, we found no direct relationship between nature dose and health and wellbeing outcomes. Instead, we found that nature connection (specifically, NR-Self) moderated the relationship between duration of nature exposure and mental wellbeing outcomes wherein individuals with a stronger connection to nature were less likely to exhibit symptoms of depression, stress, and anxiety, regardless of how long they spent in nature, while individuals with a weaker connection to nature were more likely to show more symptoms, with an increasing duration spent in nature. The lack of a direct relationship between nature dose measures and beneficial mental wellbeing outcomes was surprising, as these relationships were found in a Brisbane study [63]. Moreover, direct, multi-sensory exposure to nature has been shown to promote positive mental wellbeing outcomes via pathways that involve physical activity and social support. Indeed, visual greenery in vegetation, the scent of flowers and tree oils, and the songs of birds have all been shown to improve mental wellbeing [47,71,72] through various human senses [73]. Similarly, living near green spaces promotes a higher frequency and intensity of exercise [74,75], which in turn improves mental wellbeing [76], and by serving as places where local communities interact socially, strengthening social support that ameliorates loneliness and improves wellbeing [77,78]. However, when summed across different types of nature exposure ranging from direct (time spent in parks and on gardening), indirect (view of nature through windows) and incidental (time in nature as part of work), an average Singapore resident only had half the amount of exposure (25.8 h per week) compared to those living in Australia (52.3 h per week [63]) and the United Kingdom (57.3 h per week [30]).

Our results show that the relationship between nature exposure and wellbeing might vary depending on city context, and this is an important finding with policy and programming implications. The finding of an interaction between nature dose and connection to nature on mental wellbeing outcomes contrasts with several prior studies, where increased durations were found to be directly associated with a decrease in depression [29], and where nature-based activities were associated with lower anxiety levels compared to indoor activities [79]. Conversely, our finding that people with a higher connection to nature had fewer mental wellbeing symptoms aligns with research showing that people with stronger nature relatedness (and other similar psychological constructs such as nature connectivity, nature connectedness) have lower levels of anxiety [80] and improved subjective wellbeing [81,82,83]. Viewed holistically, this particular result indicates that strengthening how strongly people identify with the natural environment (ecological identity; NR-Self) could be important for promoting mental wellbeing benefits. Indeed, nature relatedness, or the cultural and social differences in how people perceive and construe their connection with the natural world [84], could motivate interactions with nature and enhance wellbeing [85]. In this regard, Singapore differs from many other places where such studies have been conducted. The city-state is situated in tropical south-east Asia (non-European setting), and where citizens embody a strong national identity and culture where green is integrated into daily living [86] and widely considered key to quality of life [87]. Relatedly, climate conditions unique to Singapore’s equatorial position such as higher average temperatures and humidity and regular monsoonal rainfall could also be physically uncomfortable for people to spend long durations in outdoor green spaces. These reasons could be why we found that NR-Self (*ecological identity*) but not NR-Experience (*familiarity with nature*) was associated with positive wellbeing outcomes, while other studies found that it was NR-Experience but not NR-Self that was associated with lower levels of anxiety [80,88]. It would be interesting to conduct a longitudinal study in Singapore to investigate how peoples’ construction of nature and connection with different types of nature change over time.

Our finding that people with a weaker connection to nature tended to have more symptoms of stress, depression, and anxiety with increasing duration spent in nature was also unexpected. Perhaps this subgroup comprised people who are generally disinterested or uncomfortable with spending time in nature, and prefer urban environments, modern comforts, or indoor recreational activities [89]. Alternatively, duration spent in nature could be a proxy for other activities such as physical exercise. As such, the positive correlation between higher levels of stress, depression, and anxiety with longer durations of time spent in nature could indicate a coping strategy (of increased engagement in physical exercise) that some individuals take to manage their stress levels [90]. Nonetheless, further investigation is required to understand how weakly connected individuals interact with nature, their perceptions of such interactions, and the activities that they engage in that benefit their health and wellbeing.

One strength of our study was our attempt to account (statistically) for a potential reverse pathway by having a covariate representing whether poor health impeded certain individuals from spending time in nature. We do so because individuals clinically diagnosed with anxiety or depression tend to have reduced overall physical activity levels when compared to healthy controls [90,91]. We found that, when compared to people whose poor health impeded their use of green spaces, the group reporting that poor health did not impede their use of green space had lower levels of depression, stress, and anxiety, and were less likely to be treated for high blood pressure. However, given that we did not find a significant relationship between nature dose and wellbeing, but a relationship moderated by one’s connection to nature, it is possible that the widely reported positive associations between nature exposure and health may be caused in part by healthier and more nature-connected people simply using green spaces more. Future studies could take longitudinal approaches that track people’s nature exposure and health outcomes at regular temporal intervals in a methodologically more rigorous manner than the cross-sectional study reported here.

We found no significant relationship between nature intensity (i.e., the quality of the natural environment) and mental wellbeing, despite some studies suggesting that more biodiverse environments are associated with positive health outcomes [30,92,93,94]. Comparing among studies that used tree cover as a measure of nature intensity, only one study echoed our findings [95], while others found that tree cover was associated with other wellbeing outcomes such as greater reflection, continuity with the past and attachment [26], and personal wellbeing [96]. Given that the availability of nature in Singapore is arguably saturated, since green cover is equitably distributed [97], predominantly publicly accessible, and present all year round due to its tropical (non-temperate) geolocation, it could be that other indices of nature quality are required to better distinguish between the ecological complexity in tropical natural ecosystems. Perhaps a more direct measure such as visual complexity of elements in the landscape, including their shapes and how these are arranged in space [98], might prove to be more relevant in the context of mental wellbeing [99]. Alternatively, it could be cultural or between-individual differences in how a person connects to, values, and perceives nature [100], measurements that were not captured by “tree cover”, that best deliver wellbeing benefits. For example, the bulk of nature–health studies have been conducted in European and North American settings, and those cultures may simply differ in the way they connect to, and value, nature when compared to a tropical Asian setting. As such, fundamental social, cultural, and geophysical differences between cities could impact the quantity and quality of nature dose experienced by people. Nonetheless, our measures of nature dose are self-reported, and likely less comprehensive than those obtained via geo-tracking individuals during an actual green space visit [101] or across several days [102]. We also focused our measures of exposure to nature on green space visits. While this is the predominant type of urban green space in Singapore, time spent on gardening can also be an important means of meaningful contact with nature for many, delivering substantial human health benefits [103].

We found no significant relationship between exposure to nature and both physical health outcomes (high blood pressure and diabetes). This was unexpected for diabetes, a health outcome that is highly dependent on diet and lifestyle [104,105], since having active lifestyles and optimal weights are protective against type-2 diabetes [106]. Given that living near green spaces promotes a higher frequency and intensity of exercise [74,107,108], with some groups (e.g., women) also having a lower BMI [109], we expected some measures of nature dose to be associated with a lower risk of diabetes. Our findings therefore differed from those who found that greater exposure to nature correlated with a lower risk of type-2 diabetes [110]. Of interest was that social cohesion was positively associated with diabetes, suggesting that there might be socio-cultural influences that affect food consumption patterns in ways detrimental to personal health, such as the consumption of staples high in carbohydrates [111]. We did find, however, that age and body mass index were positively associated with an increase in likelihood of receiving treatment for high blood pressure and diabetes, which aligns with other adult- [18,112,113] and child-population health studies [114,115], even after controlling for socio-economic factors. Our findings on high blood pressure as a health outcome adds another datapoint to the current state of mixed findings wherein a reduction in blood pressure (in response to exposure to nature) was reported in some studies [76,116,117] but not others [77,118]. Such mixed findings could be an artefact of our choice of response variable, where the occurrence of whether an individual was receiving treatment for diabetes or high blood pressure could be under-reported because of a fear or reluctance to report the truth, or that there are undiagnosed cases of diabetes and high blood pressure in our sample [119,120]. Future studies should consider conducting long-term studies on clinically diagnosed individuals, or stratify the sample so that it approximates the national population of people diagnosed with diabetes/high blood pressure, to better follow the relationships between changes in nature exposure and impact on physical health status.

## 5. Conclusions

We assessed the relationships between direct exposure to nature (frequency, duration, and intensity) and three mental wellbeing (depression, stress and anxiety) and two physical health (high blood pressure, diabetes) outcomes. In this context of a densely populated tropical city, we found that the relationship between duration of green space visits and mental wellbeing outcomes is moderated by nature connection. People with a stronger connection to nature had lower levels of depression, stress, and anxiety (regardless of duration of nature dose), while those with a weaker connection to nature but who spent longer durations in nature were more likely to be depressed, stressed, and anxious. We did not find a relationship between nature dose and incidence of high blood pressure and diabetes, nor a relationship between quantity and quality of green space with health and wellbeing outcomes. Viewed holistically, our results highlight a complex relationship between nature dose and health and wellbeing benefits that could vary from city to city driven by local social, cultural, and geophysical differences. Thus, locally relevant evidence, strategies, and policies are necessary to connect people to nature in ways that benefit their health and wellbeing.

## Figures and Tables

**Table 1:** Description of the predictor variables specified in each of the five global models where response variables are depression, anxiety, stress, high blood pressure, and diabetes.

Variable	Description
Age (linear)	Respondents provided their age in years.
Personal income (linear)	Respondents selected from 16 income brackets *. For analysis purposes, the mid- point of each income bracket was used and treated as a continuous variable.
Gender (categorical)	Female or male.
Ethnic group (categorical)	Respondents selected from: Chinese, Malay, Indian, Eurasian, Others. For analysis purposes, these were later aggregated to two categories: ethnic majority (Chinese) and minorities (Malay, Indian, Eurasian, Others).
Education (categorical)	Respondents indicated their highest formal education by selecting from 10 catego- ries *. For analysis purposes, these were later aggregated into two categories: No bachelor’s degree and bachelor’s degree and higher.
Number of workdays per week (linear)	The number of days the respondent works in an average week.
Physical activity (linear)	Number of days in the last week where the respondent engaged in vigorous physi- cal activity.
Body mass index (linear)	Respondent’s body mass index (BMI), calculated as: weight in kilograms divided by height in metres squared.
Social cohesion (linear)	A measure of a respondent’s perceptions of social cohesion as derived from three questions *, with higher scores representing stronger social cohesion.
Ability (or inability) to access green space be- cause of poor health (binary)	Respondents indicated the extent to which poor health prevents them from spend- ing time in outdoor green spaces. Respondents selected from: never; sometimes; of- ten; most of the time. For analysis purposes, these were later aggregated into two categories: unable to access green space because of poor health (responses of “of- ten” and “most of the time”) and able to access green space because of poor health (responses of “never” and “sometimes”).
NR-Self (linear)	A measure of a respondent’s ecological identity as derived from the nature related- ness affective sub-scale *, with higher scores representing stronger NR-Self.
NR-Experience (linear)	A measure of a respondent’s familiarity with nature as derived from the nature re- latedness experiential sub-scale *, with higher scores representing stronger NR-Ex- perience.
Frequency of green space visits (linear)	Self-reported number of visits to public outdoor green spaces in the past year.
Duration of green space visits (linear)	Self-reported average number of hour(s) spent during each visit to public outdoor green spaces in the week prior to completing the survey.
Nature intensity (linear)	Area of tree canopy within the most vegetated outdoor green space visited by each respondent, and the proportion of that which is human-managed.

* See Oh et al. [63] for the full list of response options or statements.

**Table 2:** The relationship between mental wellbeing outcomes (i.e., depression, stress, and anxiety), socio-demographic covariates, and nature exposure predictor variables. Hessian condition number (Cond. H) and estimated parameter coefficients are presented from the CLMMs, with standard error in brackets; negative coefficients indicate that prevalence of depression, stress, and anxiety are lower with higher values of predictor variables. Table cells shaded in grey (with bold numbers) represent significant predictor variables (*p*-value ≤ 0.05). An asterisk represents an interaction between two predictor variables. The estimated parameter coefficients and confidence intervals for categorical factors are presented relative to a comparative base factor level: Education: no bachelor’s degree; gender: male; ethnicity: majority Chinese; inability to access green space because of poor health).

Predictor Variables	Depression	Stress	Anxiety
	Cond. H = 2.6 × 10^3^	Cond. H = 2.3 × 10^3^	Cond. H = 8.6 × 10^1^
**Age**	**−0.45 (0.05)**	**−0.28 (0.04)**	**−0.30 (0.03)**
**Personal Income**	−0.08 (0.06)	**−0.09 (0.05)**	−0.04 (0.04)
**Education (bachelor’s degree)**	−0.06 (0.10)	0.03 (0.08)	−0.07 (0.07)
**Gender (female)**	−0.08 (0.10)	0.11 (0.08)	0.04 (0.06)
**Ethnicity (minorities)**	**−0.24 (0.11)**	**−0.20 (0.09)**	**−0.15 (0.08)**
**Number of work days**	−0.01 (0.05)	0.04 (0.04)	−0.01 (0.03)
**Body Mass Index**	−0.01 (0.05)	−0.03 (0.04)	0.01 (0.03)
**Physical activity**	0.03 (0.05)	**0.08 (0.04)**	**0.07 (0.03)**
**Duration of green space visit**	**1.17 (0.44)**	**0.90 (0.35)**	0.06 (0.04)
**Frequency of green space visits**	−0.32 (0.43)	−0.15 (0.34)	**−0.10 (0.04)**
**Average tree cover**	−0.02 (0.05)	−0.01 (0.04)	−0.01 (0.03)
**Proportion of tree cover that is managed**	0.03 (0.11)	0.09 (0.09)	−0.02 (0.08)
**NR_Self**	**−0.21 (0.10)**	−0.07 (0.08)	**−0.08 (0.04)**
**NR_Experience**	−0.08 (0.06)	−0.07 (0.05)	−0.06 (0.04)
**Social Cohesion**	**−0.18 (0.05)**	−0.07 (0.04)	0.01 (0.03)
**Ability to access green space**	**−0.69 (0.13)**	**−0.61 (0.11)**	**−0.56 (0.09)**
Duration of green space visits * NR_Self	**−0.30 (0.12)**	**−0.23 (0.10)**	**−0.10 (0.04)**
Frequency of green space visits * NR_Self	0.06 (0.12)	0.01 (0.10)	0.02 (0.05)
Duration of green space visits * NR_ Experience	0.08 (0.07)	0.06 (0.06)	0.08 (0.05)
Frequency of green space visits * NR_ Experience	0.04 (0.06)	0.06 (0.05)	−0.01 (0.04)

**Table 3:** The relationship between physical health outcomes (high blood pressure and diabetes), socio-demographic covariates, and nature exposure predictor variables. The Nagelkerke/Crag and Uhler’s pseudo R^2^ and estimated parameter coefficients are presented from the negative binomial GLMs, with standard error in brackets. Table cells shaded in grey (with bold numbers) represent significant predictor variables (*p*-value ≤ 0.05). An asterisk represents an interaction between two predictor variables.

Predictor Variables	High Blood Pressure	Diabetes
	Pseudo R^2^ = 0.35	Pseudo R^2^ = 0.21
**Age**	**1.35 (0.13)**	**0.91 (0.16)**
**Personal Income**	0.17 (0.12)	−0.06 (0.18)
**Education (bachelor’s degree)**	−0.35 (0.22)	−0.59 (0.33)
**Gender (female)**	−0.19 (0.21)	−0.54 (0.30)
**Ethnicity (minorities)**	−0.53 (0.28)	−0.24 (0.38)
**Number of work days**	0.01 (0.11)	−0.11 (0.14)
**Body Mass Index**	**0.77 (0.10)**	**0.49 (0.13)**
**Physical activity**	0.10 (0.10)	**−0.37 (0.19)**
**Duration of green space visit**	−0.03 (0.10)	−0.07 (0.18)
**Frequency of green space visits**	0.02 (0.11)	−0.22 (0.22)
**Average tree cover**	0.05 (0.10)	0.15 (0.13)
**Proportion of tree cover that is managed**	−0.27 (0.25)	−0.17 (0.35)
**NR_Self**	−0.21 (0.13)	−0.12 (0.18)
**NR_Experience**	0.09 (0.13)	0.02 (0.19)
**Social Cohesion**	0.13 (0.11)	**0.36 (0.17)**
**Ability to access green space**	**−0.80 (0.26)**	−0.32 (0.37)
Duration of green space visits * NR_Self	0.06 (0.15)	0.14 (0.21)
Frequency of green space visits * NR_Self	−0.06 (0.13)	0.04 (0.24)
Duration of green space visits * NR_Experience	−0.01 (0.16)	−0.16 (0.25)
Frequency of green space visits * NR_Experience	−0.04 (0.13)	0.05 (0.23)

**Table 4:** The relationship between whether a person was able or unable to access outdoor green spaces because of poor health, and five socio-demographic predictor variables. The Nagelkerke/Crag and Uhler’s pseudo R^2^ and estimated parameter coefficients are presented from the GLM, with standard error in brackets. Table cells shaded in grey (with bold numbers) represent significant predictor variables (*p*-value ≤ 0.05). The estimated parameter coefficients and confidence intervals for categorical factors are presented relative to a comparative base factor level: Education: no bachelor’s degree; gender: male; ethnicity: majority Chinese).

Predictor Variables	Estimated Coefficient (Standard Error)
	Pseudo R^2^ = 0.01
**Age**	**0.20 (0.08)**
**Personal Income**	0.01 (0.09)
**Education (Bachelor’s degree)**	−0.04 (0.17)
**Gender (Female)**	−0.04 (0.15)
**Ethnicity (Minorities)**	−0.26 (0.17)

## Data Availability

Upon acceptance of the manuscript, the data will be publicly archived and made available.

## Supplementary Materials

The following are available online at https://www.mdpi.com/article/10.3390/ijerph181910149/s1, Table S1: The average score and standard error for each of the three mental health outcome (depression, stress and anxiety) and two nature connection sub-scales (NR-Self, NR-Experience) as used in the GLM analyses., Table S2: The relationship between depression as a health outcome, socio-demographic covariates and nature exposure predictor variables., Table S3: The relationship between anxiety as a health outcome, socio-demographic covariates and nature exposure predictor variables., Table S4: The relationship between stress as a health outcome, socio-demographic covariates and nature exposure predictor variables., Table S5: The relationship between two health outcomes (the response variables) of depression and high blood pressure, socio-demographic covariates, and nature experience predictor variables.
