# Supplementary material for: Health and Wellbeing Benefits from Nature Experiences in Tropical Settings Depend on Strength of Connection to Nature
Source: Int J Environ Res Public Health. 2021 Sep 27;18(19):10149. doi: 10.3390/ijerph181910149 (PMC8507985; doi:10.3390/ijerph181910149)
Supplement: Supplementary file 1 [file ijerph-18-10149-s001.zip › ijerph-1383750-supplementary.pdf]

Supplementary Material S1

Table S1: The average score and standard error for each of the three mental health outcome (depression, stress and anxiety) and two nature connection sub-scales (NR-Self, NR-Experience) as used in the GLM analyses.

Variable	Mean	Standard Error
Depression	13.3	0.14
Stress	13.7	0.13
Anxiety	12.5	0.12
NR-Self	3.50	0.01
NR-Experience	3.07	0.02

Table S2: The relationship between depression as a health outcome, socio-demographic covariates and nature exposure predictor variables. We present and compare estimated parameter coefficients for two sets of analyses (in addition to the CLMM reported in the manuscript), with standard error in brackets. The first set of GLM analyses contain all the predictor variables used in the CLMM. Negative coefficients indicate that the prevalence of depression are lower with higher values of predictor variables. Table cells shaded in grey (with bold numbers) represent significant predictor variables (p-value ≤ 0.05).

	CLMM	GLM Negative Binomial
**Age**	**-0.45 (0.05)**	**-0.10 (0.01)**
**Personal Income**	-0.08 (0.06)	-0.01 (0.01)
**Education (Bachelor's degree)**	-0.06 (0.10)	-0.02 (0.02)
**Gender (Female)**	-0.08 (0.10)	-0.02 (0.02)
**Ethnicity (Minorities)**	**-0.24 (0.11)**	**-0.06 (0.02)**
**Number of work days**	-0.01 (0.05)	0.00 (0.01)
**Body Mass Index**	-0.01 (0.05)	0.00 (0.01)
**Physical activity**	0.03 (0.05)	0.00 (0.01)
**Duration of greenspace visit**	**1.17 (0.44)**	**0.03 (0.01)**
**Frequency of greenspace visits**	-0.32 (0.43)	**-0.02 (0.01)**
**Average tree cover**	-0.02 (0.05)	-0.01 (0.01)
**Proportion of tree cover that is managed**	0.03 (0.11)	0.00 (0.02)
**NR_Self**	**-0.21 (0.10)**	**-0.03 (0.01)**
**NR_Experience**	-0.08 (0.06)	-0.01 (0.01)
**Social Cohesion**	**-0.18 (0.05)**	**-0.03 (0.01)**
**Ability to access greenspace**	**-0.69 (0.13)**	**-0.15 (0.03)**
**Duration of greenspace visits* NR_Self**	**-0.30 (0.12)**	**-0.03 (0.01)**
**Frequency of greenspace visits* NR_Self**	0.06 (0.12)	0.01 (0.02)
**Duration of greenspace visits*NR_Experience**	0.08 (0.07)	0.01 (0.02)
**Frequency of greenspace visits* NR_Experience**	0.04 (0.06)	0.00 (0.01)

Table S3: The relationship between anxiety as a health outcome, socio-demographic covariates and nature exposure predictor variables. We present and compare estimated parameter coefficients for two sets of analyses (in addition to the CLMM reported in the manuscript), with standard error in brackets. The first set of GLM analyses contain all the predictor variables used in the CLMM. Negative coefficients indicate that the prevalence of anxiety are lower with higher values of predictor variables. Table cells shaded in grey (with bold numbers) represent significant predictor variables (p-value ≤ 0.05).

	**CLMM**	**GLM Negative Binomial**
**Age**	**-0.30 (0.03)**	**-0.09 (0.01)**
**Personal Income**	-0.04 (0.04)	0.00 (0.01)
**Education (Bachelor's degree)**	-0.07 (0.07)	-0.03 (0.02)
**Gender (Female)**	0.04 (0.06)	0.01 (0.02)
**Ethnicity (Minorities)**	**-0.15 (0.08)**	**-0.04 (0.02)**
**Number of work days**	-0.01 (0.03)	-0.01 (0.01)
**Body Mass Index**	0.01 (0.03)	0.00 (0.01)
**Physical activity**	**0.07 (0.03)**	0.02 (0.01)
**Duration of greenspace visit**	0.06 (0.04)	**0.02 (0.01)**
**Frequency of greenspace visits**	**-0.10 (0.04)**	**-0.03 (0.01)**
**Average tree cover**	-0.01 (0.03)	-0.01 (0.01)
**Proportion of tree cover that is managed**	-0.02 (0.08)	-0.02 (0.02)
**NR_Self**	**-0.08 (0.04)**	**-0.04 (0.01)**
**NR_Experience**	-0.06 (0.04)	0.00 (0.01)
**Social Cohesion**	0.01 (0.03)	0.02 (0.01)
**Ability to access greenspace**	**-0.56 (0.09)**	**-0.18 (0.03)**
**Duration of greenspace visits* NR_Self**	**-0.10 (0.04)**	**-0.03 (0.01)**
**Frequency of greenspace visits* NR_Self**	0.02 (0.05)	0.01 (0.01)
**Duration of greenspace visits*NR_Experience**	0.08 (0.05)	0.02 (0.01)
**Frequency of greenspace visits* NR_Experience**	-0.01 (0.04)	0.00 (0.01)

Table S4: The relationship between stress as a health outcome, socio-demographic covariates and nature exposure predictor variables. We present and compare estimated parameter coefficients for two sets of analyses (in addition to the CLMM reported in the manuscript), with standard error in brackets. The first set of GLM analyses contain all the predictor variables used in the CLMM. Negative coefficients indicate that the prevalence of stress are lower with higher values of predictor variables. Table cells shaded in grey (with bold numbers) represent significant predictor variables (p-value ≤ 0.05).

	CLMM	GLM Negative Binomial
**Age**	**-0.28 (0.04)**	**-0.07 (0.01)**
**Personal Income**	**-0.09 (0.05)**	-0.01 (0.01)
**Education (Bachelor's degree)**	0.03 (0.08)	0.00 (0.02)
**Gender (Female)**	0.11 (0.08)	0.02 (0.02)
**Ethnicity (Minorities)**	**-0.20 (0.09)**	**-0.06 (0.02)**
**Number of work days**	0.04 (0.04)	0.01 (0.01)
**Body Mass Index**	-0.03 (0.04)	0.00 (0.01)
**Physical activity**	**0.08 (0.04)**	-0.01 (0.01)
**Duration of greenspace visit**	**0.90 (0.35)**	**0.02 (0.01)**
**Frequency of greenspace visits**	-0.15 (0.34)	**-0.03 (0.01)**
**Average tree cover**	-0.01 (0.04)	-0.01 (0.01)
**Proportion of tree cover that is managed**	0.09 (0.09)	0.02 (0.02)
**NR_Self**	-0.07 (0.08)	-0.01 (0.01)
**NR_Experience**	-0.07 (0.05)	-0.02 (0.01)
**Social Cohesion**	-0.07 (0.04)	-0.01 (0.01)
**Ability to access greenspace**	**-0.61 (0.11)**	**-0.15 (0.03)**
**Duration of greenspace visits* NR_Self**	**-0.23 (0.10)**	**-0.03 (0.01)**
**Frequency of greenspace visits* NR_Self**	0.01 (0.10)	0.00 (0.01)
**Duration of greenspace visits*NR_Experience**	0.06 (0.06)	0.01 (0.01)
**Frequency of greenspace visits* NR_Experience**	0.06 (0.05)	0.01 (0.01)

Supplementary Material S2

We emulated as closely as possible the analyses in Shanahan et al. (2016). We used binomial generalised linear models for both health outcomes, depression and high blood pressure. Specifically, both response variables were treated as a binary measure where 0 = No depression (or not receiving treatment for high blood pressure); 1 = Mild/worse depression (or receiving treatment for high blood pressure). While the three measures of nature dose were uncorrelated (VIF < 3), we nonetheless generated four predictor model sets for each health response as per Shanahan et al. (2016), and an additional fifth model set with all three measures of nature dose. These model sets were: (i) all socio-demographic variables (but excluding frequency, duration, and intensity of nature experiences); (ii) all socio-demographic variables plus duration of nature experiences; (iii) all socio-demographic variables plus frequency of nature experiences; (iv) all socio-demographic variables plus nature intensity; and (v) all socio-demographic variables plus duration, frequency and intensity of nature experiences. The socio-demographic (predictor) variables were: age (responses were converted to the categories used in Shanahan et al. (2016) for comparability), personal income (linear), education (categorical: secondary school not completed; secondary school completed; vocational certification OR post-secondary; Bachelor's Degree; Postgraduate Degree), gender (categorical: male/female), ethnicity (categorical: Chinese majority; other ethnic minorities), number of work days (linear: number of days worked in an average week), BMI (linear: respondent's Body Mass Index), frequency of exercise (linear: self-reported number of days the respondent exercised per week), number of children (binary: presence or absence of people living in the respondent's home who were under 16 years at the time of survey) and social cohesion (linear). Two key differences from the Shanahan et al. (2016) analyses were: (i) a lack of predictor representing a measure of neighbourhood socio-economic disadvantage, because no such data existed; and (ii) BMI was not log-transformed BMI because our models met assumptions of normality by retaining it as a continuous variable.

Table S5: The relationship between two health outcomes (the response variables) of depression and high blood pressure, socio-demographic covariates, and nature experience predictor variables. Five models for each response variable are shown. Estimated parameter coefficients are presented, with standard error in brackets. Table cells shaded in grey (with bold numbers) indicates significant predictor variables (p-value ≤ 0.05). The estimated parameter coefficients and confidence intervals for categorical factors are presented relative to a comparative base factor level: Education: Secondary school not completed; Gender: Male; Ethnicity: Majority Chinese.

**Predictor Variables** / **Response Variables**	**Depression**	**High Blood Pressure**
**Model (i)**		
Age	**-0.03 (0.00)***	**0.09 (0.01)***
Personal Income	-0.10 (0.07)	0.19 (0.12)
Education (Completed secondary school)	-0.33 (0.68)	-1.48 (0.82)
Education (Vocational certification/post-secondary)	-0.37 (0.68)	-1.36 (0.81)
Education (Bachelor's Degree)	-0.35 (0.68)	-1.58 (0.83)
Education (Postgraduate Degree)	-0.48 (0.70)	**-2.07 (0.89)**
Gender (Female)	-0.15 (0.11)	-0.23 (0.21)
Ethnicity (Minorities)	**-0.28 (0.14)**	**-0.54 (0.28)**
Number of work days	-0.04 (0.06)	0.02 (0.10)
Body Mass Index	-0.01 (0.06)	**0.78 (0.10)**

Physical activity	0.00 (0.06)	0.13 (0.10)
Number of children	0.18 (0.12)	0.00 (0.22)
Connection (Nature-Relatedness)	**-0.27 (0.06)**	-0.14 (0.10)
Social Cohesion	**-0.23 (0.06)**	0.14 (0.11)
**Model (ii)**		
+ Duration of greenspace visit	0.03 (0.06)	0.02 (0.09)
**Model (iii)**		
+ Frequency of greenspace visits	-0.09 (0.06)	0.01 (0.09)
**Model (iv)**		
+ Average tree cover	-0.07 (0.06)	0.04 (0.09)
+ Proportion of tree cover that is managed	-0.09 (0.14)	-0.30 (0.24)
**Model (v)**		
+ Duration of greenspace visit	0.05 (0.06)	0.01 (0.09)
+ Frequency of greenspace visits	-0.08 (0.06)	-0.01 (0.09)
+ Average tree cover	-0.06 (0.06)	0.04 (0.10)
+ Proportion of tree cover that is managed	0.09 (0.14)	-0.30 (0.24)
